# Ulcerative colitis that developed during radiotherapy for prostate cancer, deteriorated rapidly and required emergency surgery

**DOI:** 10.1186/s40792-020-01024-3

**Published:** 2020-10-01

**Authors:** Keiji Matsuda, Yuka Okada, Yojiro Hashiguchi, Kentaro Asako, Kohei Ohno, Mitsuo Tsukamoto, Yoshihisa Fukushima, Ryu Shimada, Tsuyoshi Ozawa, Tamuro Hayama, Keijiro Nozawa, Takeo Fukagawa, Yuko Sasajima

**Affiliations:** 1 Department of Surgery, Teikyo University School of Medicine, 2-11-1 Kaga, Itabashi-ku, Tokyo, Japan; 2 Department of Pathology, Teikyo University School of Medicine, Tokyo, Japan

**Keywords:** Ulcerative colitis, Prostate cancer, Radiotherapy, PSA, Surgery

## Abstract

**Background:**

Although there are reports linking ulcerative colitis (UC) to prostate cancer (PC), those reports are of PC patients who were previously diagnosed with UC. There are no reports of the development of UC during radiotherapy. Here we describe the first case of a patient who developed UC during radiotherapy for PC. The UC progressed rapidly and required emergency surgery.

**Case presentation:**

A 61-year-old Japanese man underwent a prostate biopsy at another hospital due to a high prostate-specific antigen level and was diagnosed with PC. Goserelin and bicalutamide treatment was initiated in 2019, and intensity-modulated radiotherapy (total of 60 Gy/20 Fr) was administered in 2020. Diarrhea began during the radiotherapy and bleeding began post-radiotherapy. He was admitted to another hospital 14 days after the end of the radiotherapy, and colonoscopy revealed a deep ulcer in the colon, which led to the suspicion of UC. He was transferred to our hospital, and colonoscopy showed a widespread map-like ulcer, pseudopolyposis, and very easy bleeding in the colon. We diagnosed severe UC, and it worsened rapidly with uncontrollable bleeding, which we considered an indication for surgery. Emergency surgery (a total colectomy and ileostomy creation) was performed. The specimens confirmed an extensively spreading ulcer throughout the colon. The pathological report was UC in the active phase. The postoperative course was good.

**Conclusions:**

When a patient exhibits diarrhea while undergoing radiotherapy for PC, clinicians should be aware of the possibility of UC in addition to radiation colitis, and colonoscopy should be considered.

## Background

Ulcerative colitis (UC) is a disease that begins with diarrhea and bleeding and gradually worsens, but it sometimes progresses rapidly to the point of requiring emergency surgery [1]. Although there are clear data linking inflammatory bowel disease (IBD) to colorectal cancer [2, 3], several reports have linked IBD patients and the risk of prostate cancer (PC) [4, 5]. Prostate cancer is the most common cancer in men in the U.S. and UK [5, 6]. The effect of radiation for PC on patients’ ulcerative colitis has been studied [4, 5, 7, 8], but those reports are of PC patients who were previously diagnosed with UC. We have found no reports of a patient who developed UC after the diagnosis of PC. Here, we report the rare case of a patient who developed severe UC during radiotherapy (RT) for his PC. The UC progressed rapidly and required emergency surgery.

## Case presentation

**Fig. 1:**
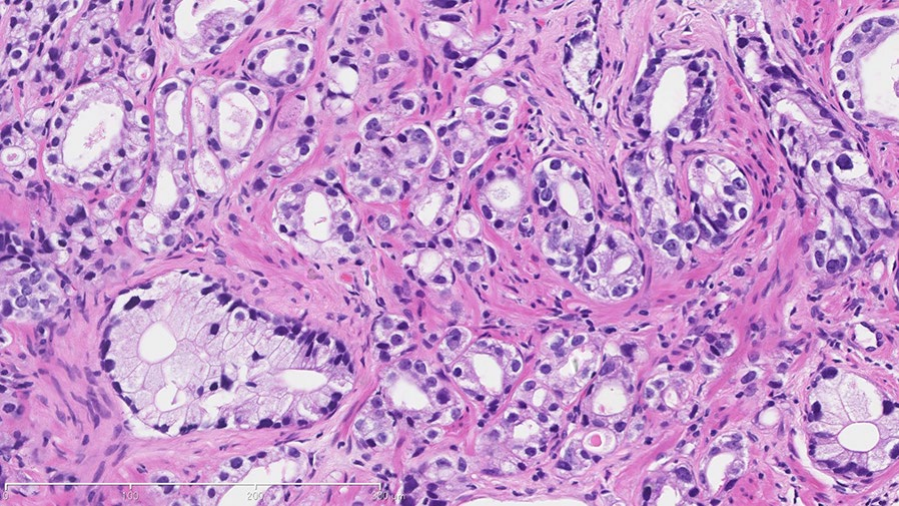
Microscopically, the biopsied specimen was composed of eosinophilic tumor cells with oval nuclei, which exhibited fused microacinar gland pattern by hematoxylin–eosin (HE) staining. The Gleason score was 4 + 3 = 7

**Fig. 2:**
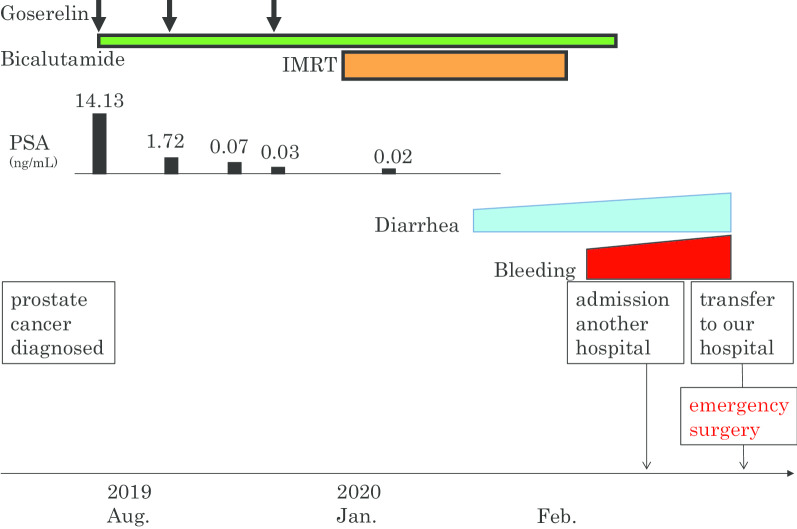
Clinical course after the diagnosis of prostate cancer

A 61-year-old Japanese man had been treated for schizophrenia and showed a high level of prostate-specific antigen (PSA, 11.68 ng/mL) at a clinic. He underwent a prostate biopsy at another hospital and was diagnosed with prostate cancer (Fig. 1). In August 2019, treatment with goserelin (subcutaneous injection) and bicalutamide (oral administration) was initiated at our hospital’s urology department. From January to February in 2020, intensity-modulated radiation therapy (IMRT; total of 60 Gy/20 Fr) was administered (Fig. 2). The patient’s PSA level went down. Diarrhea began during this IMRT period, and bleeding began after the completion of the IMRT.

At 14 days after the end of the RT, the patient was admitted to another hospital. He was hospitalized with a diagnosis of radiation colitis. He continued fasting, and an intravenous drip was given. Three days post-admission, colonoscopy revealed a deep ulcer in the colon, which led to the suspicion of UC. The next day, he was transferred to our Teikyo IBD Center (Fig. 2).

**Fig. 3:**
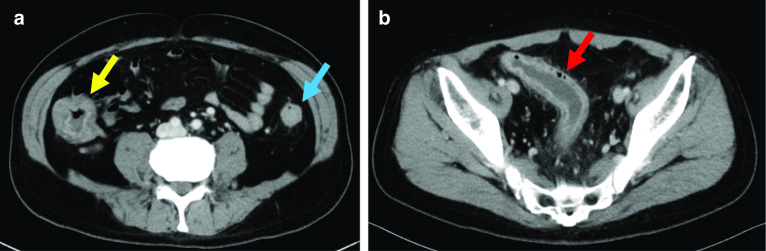
CT showed edematous and thickening of the colon [**a** ascending colon (yellow arrow) and descending colon (blue arrow), and **b** sigmoid colon (red arrow)]

**Fig. 4:**
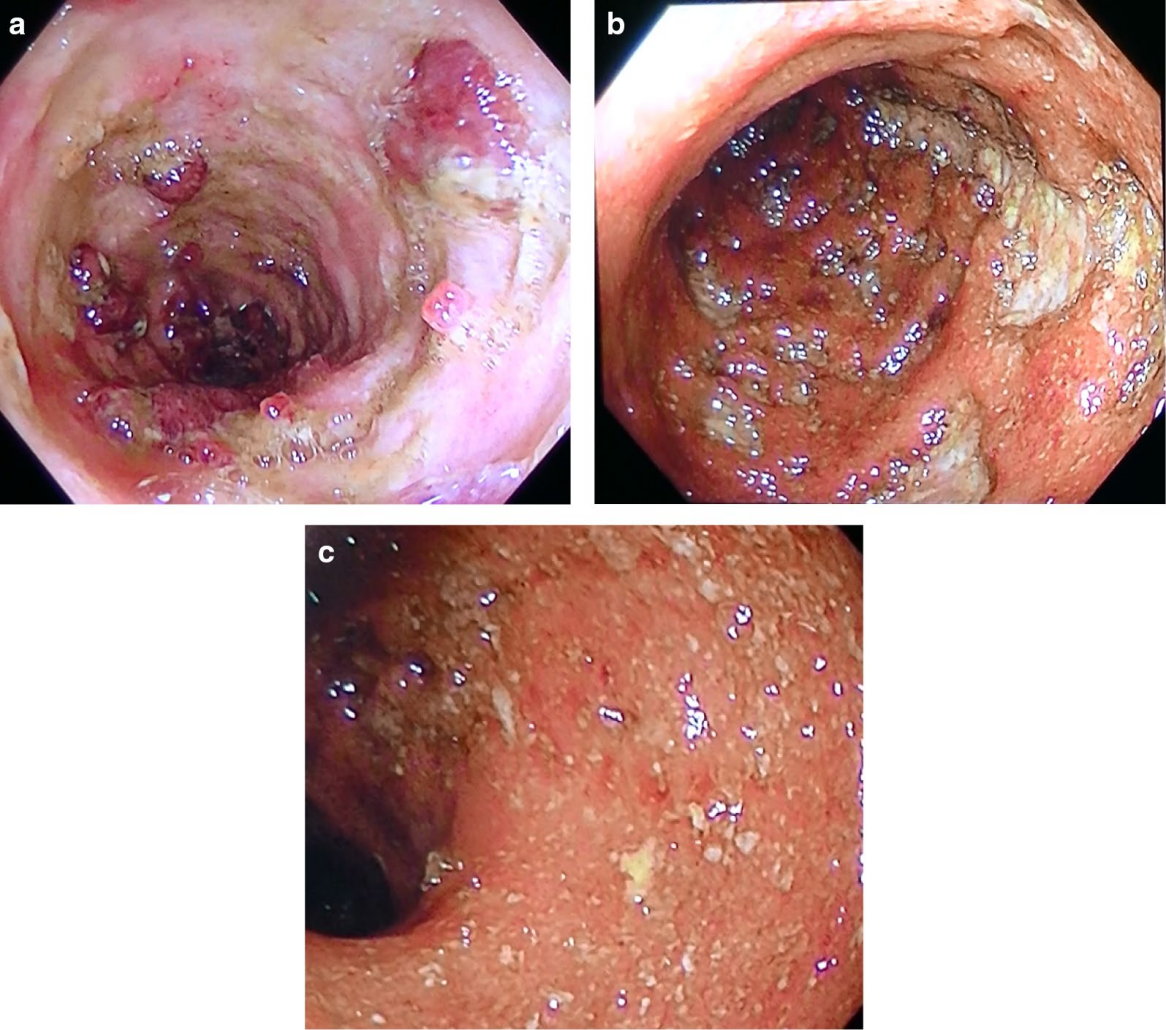
Colonoscopy performed on the day of transfer. A circumferential ulcer was found in the transverse colon (**a**). A deep ulcer was found in the sigmoid colon (**b**). Edematous inflammation with no ulcer was found in the rectum (**c**)

On physical examination, the patient's abdomen was flat and soft without tenderness or distension. The laboratory data were as follows: RBCs 356 × 10^4^/μL (low), hemoglobin 9.9 g/dL (low), WBCs 9100/μL, platelets: 32.5 × 10^4^/μL, total protein 4.7 g/dL (low), albumin 1.5 g/dL (low), and CRP 14.41 mg/dL (high). He passed bloody diarrhea and the number of stools was > 10/day. Contrast CT showed diffuse edema and wall thickening throughout the colon (Fig. 3a, b). Colonoscopy showed a widespread map-like ulcer, pseudopolyposis, and very easy bleeding in the colon but edematous inflammation with no ulcer in the rectum (Fig. 4a–c). The patient’s Disease Activity Index [9] was 11. We diagnosed with severe UC that worsened rapidly with uncontrollable massive bleeding, which was considered an indication for surgery. Emergency surgery was performed on the day of the patient's transfer to our Center.

**Fig. 5:**
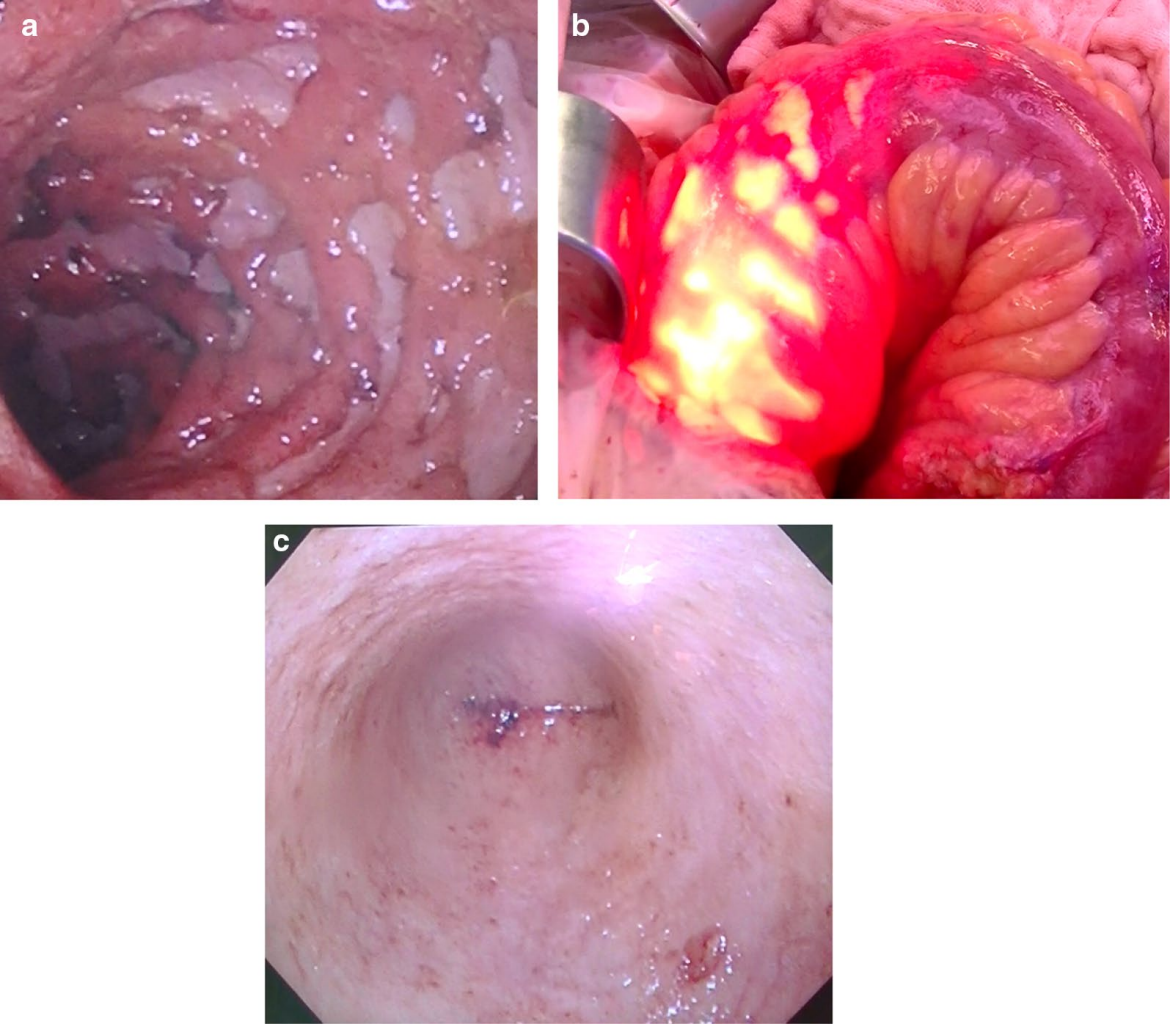
Intraoperative colonoscopy. A deep ulcer was observed in the sigmoid colon (**a**). The light from the endoscope could be seen through the ulcer area (**b**). Residual rectum showed no deep ulcer (**c**)

The surgery (total colectomy and creation of an ileostomy) was performed as follows. The abdomen was opened by a midline incision of the entire abdomen. Edema, redness, hyperemia, and thickening in the colon were observed. Intraoperative endoscopy revealed a deep ulcer in the sigmoid colon, but the rectum was slightly inflamed with no ulcer (Fig. 5a–c). A cut-off between the sigmoid colon and the rectum was selected. After transection of blood vessels, the ileocolic artery and vein were preserved and the ileum was cut-off at the terminal ileum. An ileostomy was created in the lower right abdomen.

**Fig. 6:**
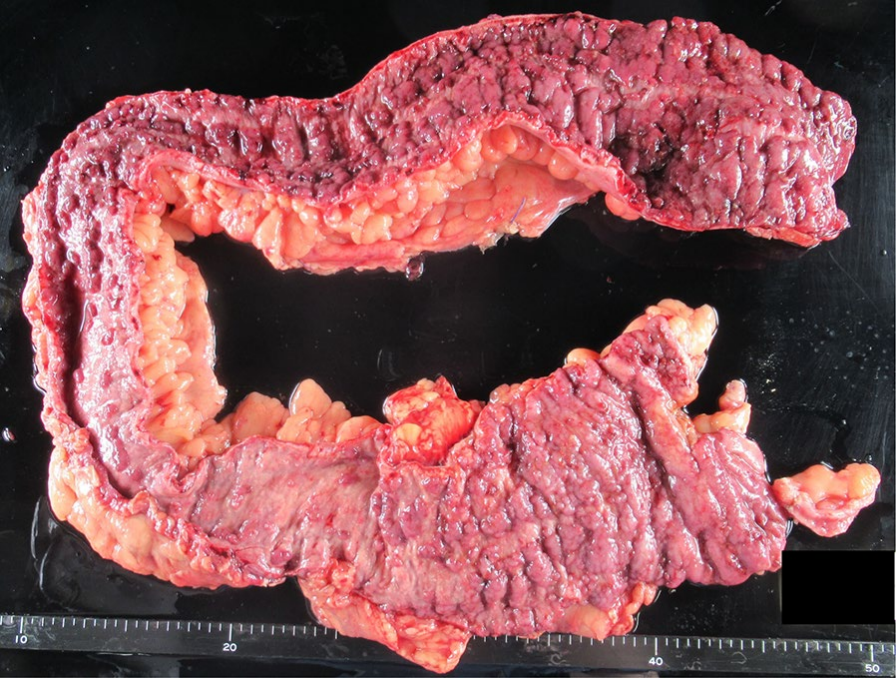
Excised specimen. Ulcers were spreading throughout the colon

**Fig. 7:**
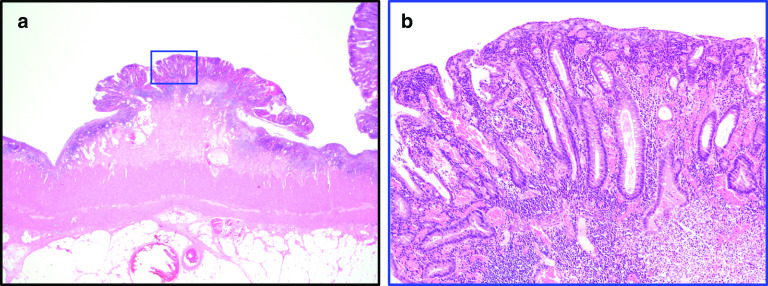
Histological findings of the transverse colon. Residual mucosal islands were located between ulcerated areas (**a** HE staining, 10×). Crypt branching and irregularity of size and shape with chronic inflammatory cells were observed in some areas (**b** HE staining, 100×)

The specimens showed an ulcer spreading extensively throughout the colon (Fig. 6). The pathological report was UC in the active phase (Fig. 7a, b). The patient’s postoperative course was good, and he was discharged 26 days after the operation.

## Discussion

This patient suffered from diarrhea during the radiotherapy for his prostate cancer, and he was thought to have radiation colitis when he was admitted to the previous hospital; however, UC developed and worsened during and after his radiotherapy. To investigate the issue of whether the radiation or the patient's PC caused his ulcerative colitis, we searched the Medline (using PubMed) for studies reporting cases of UC patients undergoing radiotherapy for PC. The search terms included (ulcerative colitis) (prostate cancer) (radiation therapy) in all fields. Our search identified 20 publications by all three search terms, 226 documents by UC and RT, and 83 documents by UC and PC. The studies that have evaluated radiotherapy for PC in UC patients are summarized in Table 1 [5, 7, 8, 10–15]. In all of these cases, the patient's UC had already been diagnosed prior to the occurrence of PC.

**Table 1:** Summary of studies assessing radiotherapy for prostate cancer in IBD patients

Author	Year	Refs	Patients	Percentage of PC in IBD pt	Radiation
Feagins et al.	2019	[7]	IBD 100 (UC 66, CD 29, IC 5)	100	Brachytherapy and/or EBRT
Mohammed et al.	2018	[5]	IBD 11 (UC 6, CD 5)	100	Brachytherapy
Kirk et al.	2017	[10]	IBD 240 (UC, CD: n.d.)	100	RT not otherwise specified
Gestaut et al.	2017	[8]	IBD 18 (UC 7, CD 2)	100	EBRT or brachytherapy
Annede et al.	2017	[11]	IBD 28 (UC 15, CD 13)	42	EBRT
Peters et al.	2006	[12]	IBD 24 (UC 17, CD 7)	100	Brachytherapy
Chen et al.	2006	[13]	IBD 52 (UC, CD: n.d.)	100	Brachytherapy and/or EBRT
Willett et al.	2000	[14]	IBD 28 (UC 18, CD 10)	25	EBRT
Grann et al.	1998	[15]	IBD 6 (UC 3, CD 3)	100	Brachytherapy

Several reports have described a correlation between IBD and the risk of PC. Men with IBD had a significantly higher incidence of PC (4.4% at 10 years) compared to controls (0.65% at 10 years) [16]. In other studies, IBD patients had significantly elevated risks for PC (relative risk: 1.70; standardized incidence ratio: 2.47) [17, 18]. A meta-analysis indicated that the presence of IBD posed a 78% increase in the risk of developing PC [19]. The underlying pathophysiology of how IBD might increase one’s risk of prostate cancer has been discussed [4]; one possibility is that the local or systemic inflammation associated with IBD may lead to prostatic inflammation, predisposing patients with IBD to the development of prostate cancer [16]. Another possibility is the translocation of pro-inflammatory bacteria from the gastrointestinal tract to the prostate by either the blood stream or the urinary tract, leading to chronic inflammation of the prostate, which, in turn, increases the risk of PC [4, 20]. Shared genetic susceptibility for prostate cancer and IBD has also been raised as a possible explanation [16].

Many studies have attempted to assess the safety of RT in patients with IBD [5, 7, 8, 11]. An examination of the correlation between pelvic RT and IBD patients showed that only the patients with a rectal IBD location or a low body mass index had experienced more severe IBD activity within or after 6 months following the RT [11]. A study assessing IBD-specific outcomes in 100 veterans with IBD revealed that the rates of IBD-associated hospitalizations and IBD-related surgeries were not significantly different between those treated with or without radiation, and there was no effect of radiation on longer-term outcomes [7]. IBD was not considered as an absolute contraindication to RT, and brachytherapy and IMRT might have less bowel toxicity compared to conventional methods of external beam RT [4]. In addition, we searched the Medline (using PubMed) for studies reporting cases of (UC and goserelin) or (UC and bicalutamide), which revealed no papers on them had been published.

We found no reports of patients who were diagnosed with UC after they developed PC or patients who developed UC during their radiation therapy. In our patient’s case, his UC symptoms began after the beginning of the radiation therapy for his PC, and the inflammation was more severe in the colon than the rectum. The following two hypotheses can be inferred for the course of this case. (1) IMRT caused an immune abnormality in the rectum, which triggered UC, and inflammation rapidly spread to the entire large intestine in a short period of time. (2) IMRT happened to coincide with the onset of UC at the same time, and IMRT was not involved in the onset of UC. This is the first report of a case of ulcerative colitis that developed during radiotherapy for prostate cancer.

## Conclusions

We report a case of UC that developed during radiation therapy for PC, which deteriorated rapidly and needed emergency surgery. When a patient with PC exhibits diarrhea, while he is undergoing radiation therapy, it is necessary to be aware of the possibility of UC in addition to radiation colitis, and colonoscopy should be considered.

## Data Availability

The authors declare that all the data in this article are available within the article.

## Abbreviations

IBD: Inflammatory bowel disease
IMRT: Intensity-modulated radiation therapy
PC: Prostate cancer
PSA: Prostate-specific antigen
RT: Radiotherapy
UC: Ulcerative colitis
